# Functional mass spectrometry imaging maps phospholipase-A2 enzyme activity during osteoarthritis progression

**DOI:** 10.7150/thno.86623

**Published:** 2023-08-21

**Authors:** Xiwei Fan, Reuben S. E. Young, Antonia Rujia Sun, Brett R. Hamilton, Udhaya Nedunchezhiyan, Ross Crawford, Stephen J. Blanksby, Indira Prasadam

**Affiliations:** 1 Centre for Biomedical Technologies, Queensland University of Technology, Brisbane, Australia.; 2 School of Mechanical, Medical & Process Engineering, Queensland University of Technology, Brisbane, Australia.; 3 Central Analytical Research Facility and School of Chemistry and Physics, Queensland University of Technology, Brisbane, Australia.; 4 Molecular Horizons and School of Chemistry and Molecular Biosciences, University of Wollongong, Wollongong, Australia.; 5 Centre for Microscopy and Microanalysis, University of Queensland, Brisbane, Australia.; 6 The Prince Charles Hospital, Orthopedic department, Brisbane, Australia.

**Keywords:** osteoarthritis, lipidomics, microenvironment, MALDI-MSI, enzyme dynamics

## Abstract

**Background:** Enzymes are central components of many physiological processes, and changes in enzyme activity are linked to numerous disease states, including osteoarthritis (OA). Assessing changes in enzyme function can be challenging because of difficulties in separating affected tissue areas that result in the homogenisation of healthy and diseased cells. Direct correlation between spatially-resolved enzyme distribution(s) and diseased cells/tissues can thus lead to advances in our understanding of OA pathophysiology. Herein, we present a method that uses mass spectrometry imaging (MSI) to visualise the distribution of lipase enzymes and their downstream lipid products in fresh bone and cartilage tissue sections. Immunohistostaining of adjacent tissue sections was then used to identify OA cells/tissues, which were then statistically correlated with molecular-level images.

**Methods:** MSI was used to image lipase enzymes, their substrates, and their metabolic products to validate enzymatic activity and correlate to OA regions determined by immunohistochemistry (IHC). Based on the modified Mankin score, six non-OA and OA patient-matched osteochondral samples were analysed by matrix-assisted laser desorption/ionisation mass spectrometry imaging (MALDI-MSI). Due to the involvement of phospholipase A2 (PLA_2_) in inflammatory pathways, explant tissues were treated with IL-1β to mimic inflammation observed in OA. Bovine explant tissues were then subject to MSI methods to observe the spatial distribution of PLA_2_.

**Results:** Compared with non-OA samples, OA samples showed an elevated level of multiple arachidonic acid (AA)-containing phospholipids (*P* < 0.001), in which the elevation in the surface and deep layer cartilage of OA tissues is correlated to elevated PLA_2_ activity (*P* < 0.001). Bovine explant tissues treated with IL-1β to mimic OA pathophysiology validated these results and displayed elevated PLA_2_ levels in OA mimic samples relative to the controls (*P* < 0.001). It was established that the PLA_2_G_2_A isoform specifically was responsible for PLA_2_ enzyme activity changes in OA tissues (*P* < 0.001).

**Conclusion:** Our results present a reliable method for imaging enzyme dynamics in OA cartilage, which sets up the foundation for future spatial enzyme dynamics in the OA field. We demonstrated that OA patients exhibit increased expression of PLA_2_G_2_A at the superficial and deep cartilage zone that degrades cartilage differently at the spatial level. A tissue-specific PLA_2_G_2_A precision inhibition may be the potential target for OA.

## Introduction

Enzymes are the catalysts responsible for many physiological events in the joints. Consequently, numerous pathologies are associated with variations in enzyme abundance, activity, specificity, or efficacy 1, 2. No single imaging modality can consistently map the distribution of enzymes within tissues while simultaneously assessing their catalytic efficiency and selectivity 1. Through the sensitive identification and high-resolution mapping of unlabelled analytes, such as regional alterations in metabolic profiles resulting from dysregulation of enzyme activity, mass spectrometry imaging (MSI) partially resolves this issue 1, 2. Traditional MSI approaches generate complicated spectra that are hampered by the poor structural elucidation of identified chemicals and the inability to link specific metabolites to distinct metabolic pathways 1. To overcome this, functional MSI (fMSI) has been developed based on depositing a reactive substrate during tissue preparation to spatially resolve unique metabolites 1, 3, which opens the possibility of going beyond simply localising biomolecules to mapping enzymatic activity.

Phospholipase A_2_ (PLA_2_) enzymes are a lipolytic enzyme superfamily that catalyses membrane phospholipid hydrolysis to create 1-lysophospholipids and free fatty acids 4. PLA_2_ is classified into three categories based on its current location, amino acid order homology, and functional characteristics: secretory, cytosolic, and Ca^2+^ non-dependent PLA_2_ 5, 6. PLA_2_ is thought to catalyse the release of arachidonic acid to produce lipid mediators of inflammation and is important in various inflammatory processes. Multiple PLA_2_ subtypes are expressed in OA chondrocytes and are affected by proinflammatory stimuli 4. However, protein abundance change does not equal functionality change due to post-modifications and the influence of other factors 7. To date, no direct measurement exists for the proinflammatory enzyme activity in OA tissue.

In the current study, we used MALDI coupled to a reflectron time-of-flight (TOF) mass spectrometer for the investigation of non-OA and OA osteochondral sections to reveal differences in the spatial enzyme dynamics of PLA_2_ by detecting arachidonic acid (AA)-containing phosphatidylinositol 38:4 (PI 38:4), phosphatidylethanolamine 38:4 (PE 38:4) and phosphatidylserine 38:4 (PS 38:4). We also deployed fMSI by depositing the stable isotope labelled substrate, 1-pentadecanoyl-2-oleoyl(D_7_)-*sn*-glycero-3-phosphocholine (PC 15:0/18:1-D_7_), to the tissue sections with subsequent monitoring of substrate-product (1-pentadecanoyl-*sn*-glycero-3-phosphocholine, LPC 15:0) ratios revealing regions of enzyme activity. Finally, an IL-1β-induced OA-mimic bovine explant *in vitro* model was used for verifying the OA-induced PLA_2_ elevation at a spatial level using high-definition MALDI-MSI.

## Methods

### Sample collection

**Figure 1:**
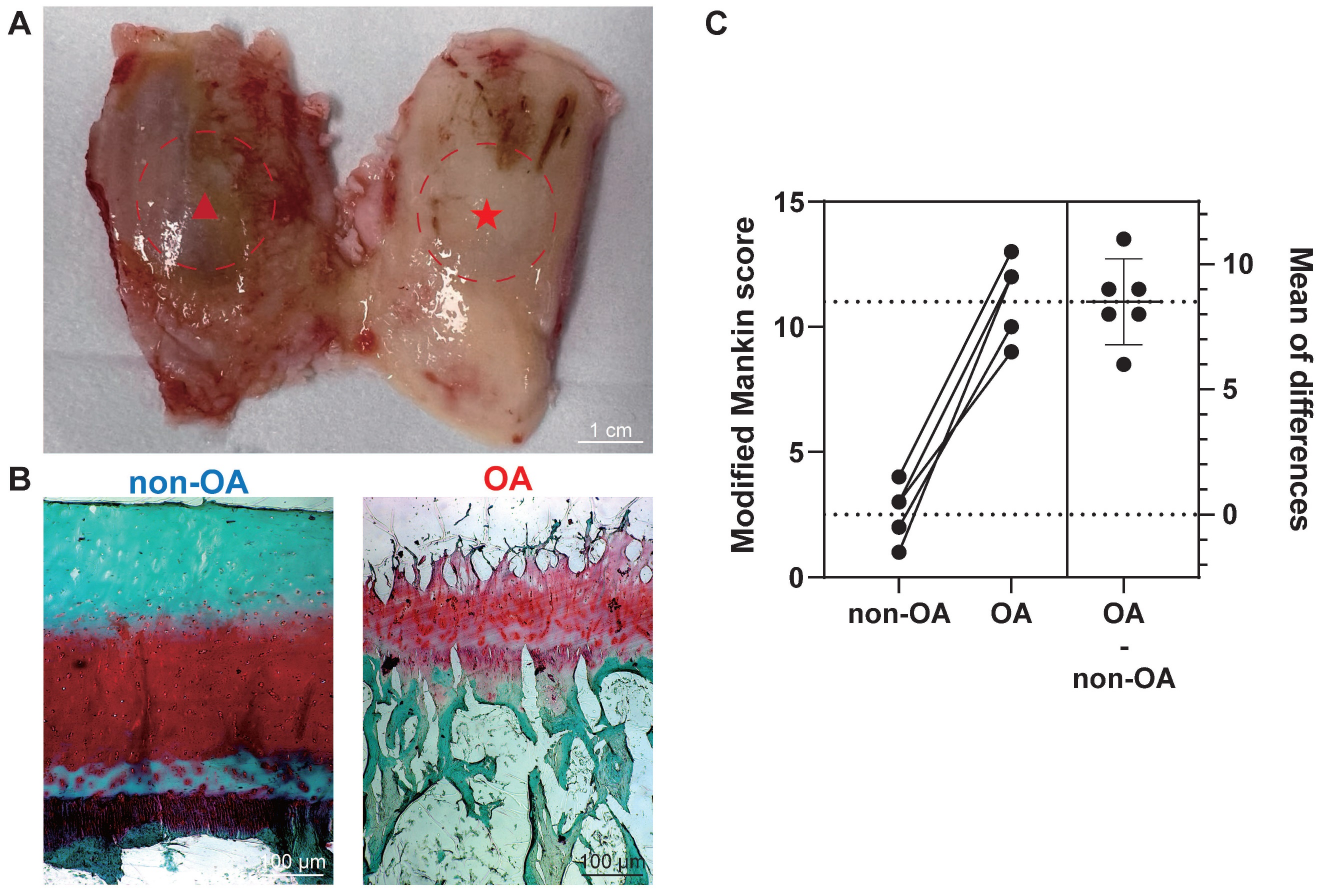
**Histopathological assessment for the section grouping based on modified Mankin score. (A)** Depiction of a femur from a total knee replacement patient, showcasing non-OA cartilage (non-OA, ★) and OA cartilage (OA, ▴). Scale bar: 1 cm. **(B)** Exemplary Safranin-O/Fast green staining utilised in histopathological evaluation. Scale bar: 100 µm. (**C**) Graphic illustrations of modified Mankin score (0-14) for histopathological examination. Data are presented as means ± standard deviation (SD) for n = 6. *P* < 0.05 was considered significant. *P < 0.05, **P < 0.01, ***P < 0.001.

Clinical specimens of the femoral condyle were obtained during total knee arthroplasty surgery and processed according to our previously established protocol 8. Ethics approval for this experiment was given by the Queensland University of Technology and St Vincent Private Hospital Ethics Committees, and all participants provided informed consent (Human ethics number: 1400001024). Patient age, gender, and body mass index (BMI) were recorded, and 1 cm x 1 cm x 1 cm cubes were cut from non-OA and OA osteochondral units (classified according to the Modified Mankin scoring system) 9 using an EXAKT 310 Diamond Band Saw (EXAKT Apparatebau GmbH - Co. Norderstedt, Germany). Detailed demographic information is provided in **Table S1.** Patients with (i) inflammatory bone disease; (ii) knee trauma; or (iii) who were using bisphosphonates or other drugs that potentially impact bone and cartilage metabolism and quality were excluded from sample collection (**Figure 1**).

### Sample sectioning and slide mounting

Samples were prepared using a previously published protocol based on the Kawamoto method 10. Specifically, the samples were immersed in liquid nitrogen on a weigh boat before embedding in a super-cryo embedding medium (SCEM, Section-lab, Yokohama, Japan). Then the sample blocks were frozen down with a hexane-dry ice mixture according to a published protocol 11. Cryosectioning methods were similar to a previously described protocol 12 whereby the CryoStar NX70 cryostat (Thermo Fisher Scientific, Waltham, USA), equipped with a D-profile tungsten carbide knife (Dorn - Hart Microedge, Loxley, USA), was used to produce 10 μm sections. Subsequently, the specimen was cooled to -30 °C and the blade to -28 °C. Next, the tissue was bonded to Cryofilm tape (3C(16UF), SECTION-LAB, Yokohama, Japan), and the blocks were cut into 1 mm x 1 mm pieces to stabilise them. The Cryofilms were then placed onto a microscope slide. Formalin-fixed, paraffin-embedded (FFPE) samples were prepared at 5 μm using a microtome previously documented in detail by our team 10.

### Safranin-O / Fast-green staining assay

Fresh-frozen samples were initially rinsed with distilled water and subsequently stained for 2 min using Weigert's iron hematoxylin working solution. A thorough 10 min wash with running tap water followed. Then, tissue sections underwent a 15 s acid ethanol immersion (or 3 quick dips), followed by a 5 min tap water rinse. After that, a 5 min fast green staining was applied. This was succeeded by a brief 10-15 s 1% acetic acid treatment and a 10 min 0.01% Safranin-O staining. Sequential dehydration and clarification of sections were then performed using 70%, 90%, and 100% ethanol, followed by xylene. Subsequently, specimens were preserved with Entellan New Mounting Medium (Merck KGaA, Darmstadt, Germany), employing an adapted version of Kawamoto's film technique tailored for high-resolution microscopy 11, 13.

### Matrix-assisted laser desorption/ionisation mass spectrometry imaging

For the spatial proteomics assay, a 250 μL solution containing 0.083 g/L sequencing-grade trypsin (pH 7.5, Roche, Basel, Switzerland) was uniformly sprayed onto bone cartilage sections with 12 layers using SunCollect Sprayer (Sunchrom, Friedrichsdorf, Germany). Based on optimisation experiments and manufacturer instructions, sequencing grade trypsin incubation was performed for 24 h at 50 °C, 98% humidity, and 1% fan strength in SunDigest Incubator (Sunchrom, Friedrichsdorf, Germany). To prepare for MALDI-MSI analysis, matrix (7 mg/mL cyano-4-hydroxycinnamic acid, CHCA) in 50% aqueous acetonitrile containing 0.2% trifluoroacetic acid (TFA) was deposited onto the tissue after digestion using a SunCollect Sprayer (Sunchrom, Friedrichsdorf, Germany) with 12 layers using line distance of 2 mm, z-position of 30 mm, a speed of approximately 695 mm/min and flow rate of 50 μL/min for layer 1, 20 μL/min for layer 2, 30 μL/min for layer 3 and 40 μL/min for all remaining layers.

fMSI was conducted following a previously published protocol 1. Briefly, 250 μL of a 100 µM solution of 1-pentadecanoyl-2-oleoyl(D_7_)-*sn*-glycero-3-phosphocholine (PC-15:0/18:1-D7) (Avanti Polar Lipids, Alabaster, USA) in a 10% (v/v) methanolic aqueous solution was sprayed on the sections using SunCollect Sprayer (Sunchrom, Friedrichsdorf, Germany) and incubated the samples for 24 h in 37 °C using a SunDigest Incubator (Sunchrom, Friedrichsdorf, Germany) with 98% humidity. To prepare for MALDI-MSI analysis, matrix (20 mg/mL norharmane in methanol) was deposited onto the tissue after digestion using a SunCollect Sprayer with 20 layers, line distance of 2 mm, z-position of 30 mm, a speed of approximately 695 mm/min, and flow rate of 40 μL/min for all layers.

A timsTOF fleX MALDI-2 (Bruker, Bremen, Germany) was used to acquire the MSI and fMSI experiments and was controlled using timsControl 3.1 and calibrated using ESI tunemix (Agilent Technologies, G1969-85000, Santa Clara, USA). Peptide imaging was conducted as a MALDI-1 (500 shots at 10 kHz) over *m/z* 800-4000 in positive mode, with funnel-1 RF 500 peak-to-peak voltage (Vpp), funnel-2 RF 450 Vpp, multipole-RF 600 Vpp, collision-RF 1500 Vpp, collision energy 10 eV, transfer time 150 μs, and pre-pulse storage of 5 μs in positive mode. The laser used customised settings by selecting Custom from the laser settings drop-down menu specifying the parameters as follows; single beam (approx. 5 μm) with beam scan set to 46 μm (X and Y) to produce a pixel size of 50 μm x 50 μm. Lipid imaging was conducted as a MALDI-1 (200 shots at 10 kHz) over *m/z* 170-1500 in negative mode, with funnel-1 RF 350 Vpp, funnel-2 RF 350 Vpp, multipole-RF 350 Vpp, collision-RF 1500 Vpp, collision energy 15 eV, transfer time 80 μs, and pre-pulse storage of 10 μs in negative mode. The laser used customised settings by selecting Custom from the laser settings drop-down menu specifying the parameters as follows; single M2 beam (approx. 5 μm) with beam scan set to 16 μm (X and Y) to produce a pixel size of 20 μm x 20 μm.

The fMSI data was conducted as a MALDI-1 (1000 shots at 10 kHz) over *m/z* 170-2000 in positive mode, with funnel 1 RF 300 Vpp, Funnel 2 RF 350 Vpp, multipole RF 400 Vpp, Collision RF 1500 Vpp, collision energy 10 eV, transfer time 60 μs, and pre-pulse storage of 10 μs in positive mode. The laser used Imaging 20 μm which includes a single beam (approx. 5μm) with beam scan set to 16 μm (X and Y) to produce a pixel size of 20 μm x 20 μm. All data were visualised using SCILS LAB MVS (version 2022b, Bruker, Bremen, Germany). The total ion current (TIC) normalisation was employed to ascertain the relative intensity of ions within designated regions of interest (ROI). Specifically, the relative intensity is quantified as a ratio of the aggregate count of species within a given area to the cumulative population of all species present within that area.

### Lipidomics analysis

Upon acquiring the samples, four patient-matched clinical specimens were dissected into osseous and cartilaginous components using a scalpel blade. Subsequently, these specimens underwent rapid immersion in liquid nitrogen to facilitate freezing. The sample blocks were placed in a freeze dryer (Scitek, Sydney, Australia) for 48 h to eliminate excess water. Once dried, the specimens were assembled within a pre-chilled grinding jar set (QIAGEN, Venlo, Netherlands), accompanied by a stainless-steel bead (QIAGEN, Venlo, Netherlands) and the whole asset was installed onto the TissueLyser II (QIAGEN, Venlo, Netherlands). Following the addition of approximately 5 mL of liquid nitrogen, the grinding jar underwent vibration within the TissueLyser II for a duration of 3min, ultimately yielding a homogenised powder.

Based on a previously published methyl-tert-butyl ether extraction (MTBE) method 14, later adapted by Young et al. 15, for tissue lipid analysis, tissue homogenate powders underwent lipid extraction. Briefly, 200 µL of methanol (with 0.01% w/v dibutyl hydroxytoluene), 20 µL of a mixture of deuterated lipid standards (SPLASH Lipid-o-mix, Avanti Polar Lipids, Alabaster, USA) and 780 µL of MTBE were added to ~20 mg of tissue homogenate in 2 mL glass vials. Vials were sealed using vial caps (PTFE septa) prior to 1 h of agitation using a vortex. Phase separation was subsequently induced using 200 µL of aqueous ammonium acetate [150 mM] prior to 30 s of further agitation (vortex) and 5 min of centrifugation (2000 × g). Ensuring the phase interface was not disturbed, organic supernatants were pipetted off and stored in clean 2 mL glass vials at -20 °C prior to mass spectrometric analyses.

Direct injection mass spectrometric analysis of lipid extracts was undertaken on an Orbitrap Elite high-resolution mass spectrometer (Thermo Scientific, Bremen, Germany), fit with a chip-based nano-electrospray source (TriVersa Nanomate, Advion, Ithaca, NY, USA). For negative polarity analysis, a chlorinated alkali buffer solution was created to both assist the deprotonation of most glycerophospholipid classes and allow for lipid-chloride adducts of phosphatidylcholine and sphingomyelin to be formed. For this, a concentrated ammoniacal buffer solution (pH 9.7) was created using 90 mg of ammonium chloride, 300 µL of aqueous ammonia (v/v 30%) and 400 µL water (LC-MS grade). A 2% methanol (LC-MS grade) solution from this stock buffer was freshly prepared before the samples were analysed. The dilute methanolic ammoniacal buffer solution was mixed 1:1 with the MTBE lipid extract solutions before injection into the mass spectrometer using 1.3 kV and 0.3 psi spray parameters. For mass spectrometry (MS^1^) analysis in the orbitrap, approximately 100 scans were acquired using a mass resolving power of 240,000 (at 400 *m/z*), an injection time of ~10 ms, and a 100-2000 *m/z* range. Subsequently, data-independent tandem mass spectrometry (MS^2^) analysis of analytes was undertaken. Using an isolation window of 1 Da, an injection time of 250 ms, and applying a normalised collision energy of 28 in the linear ion trap, the range of 650-950 *m/z* was sequentially stepped through, with mass analysis of each event occurring in the orbitrap. An in-house lipid database was generated based on theoretical *m/z* values calculated to 4 decimal places. This database contains logical sum composition assignments from lipid classes, including ether-linked phosphatidylcholine (PC O-), phosphatidylethanolamine (PE), ether-linked phosphatidylethanolamine (PE O-), phosphatidylserine (PS), phosphatidylglycerol (PG), phosphatidylinositol (PI), sphingomyelin (SM), lysophosphatidylcholine (LPC), lysophosphatidylethanolamine (LPE), lysophosphatidylserine (LPS), lysophosphatidylglycerol (LPG) and lysophosphatidylinositol (LPI), which are commonly observed in mammalian cells and tissues. MS^1^ data were used to assign lipid species at the sum composition level using a delta-ppm threshold of <5 ppm, afforded by 240,000 mass resolving power at 400 *m/z*. Sum composition species were subsequently validated, and fatty acyl substituents were assigned by investigating negative polarity MS^2^ spectra and product ions following the in-depth lipid fragmentation pathways described by Murphy and Axelsen 16.

### Bottom-up proteomics assay

Protein digestion from fresh-frozen sections was carried out according to the S-Trap Micro Spin Column digestion 17. Tissue was lysed by adding 25 µL sodium dodecyl sulphate (SDS) lysis buffer (10% SDS, 100 mM Tris) to 25 µg of the sample before dithiothreitol (DTT, 500 mM in water) was added to a final concentration of 20 mM, the resultant mixture was heated for 60 min at 70 ℃. After cooling to room temperature, the proteins in the sample were alkylated using iodoacetamide (IAA) at a final concentration of 40 mM. The samples were incubated in the dark for 30 mins and then sonicated to remove nucleic acids. Following centrifugation for 8 min at 13,000 g, 2.5 µL of 12% phosphoric acid and 165 µL of S-Trap binding solution (90% ethanol, 100 mM final Tris) was added. The mixed and acidified lysate/S-Trap buffer was placed into an S-Trap spin column and spun using a bench-top centrifuge at 4,000 g for 1 min. The flow-through was discarded. The protein was further cleaned by washing three times with 150 µL S-Trap binding buffer. Subsequently, 1 µg of trypsin in 50 µL of 50 mM Ammonium bicarbonate (ABC) pH 8.0 was added to the top of the protein trap. Then the spin column was loosely capped, and the samples were incubated in clean tubes for 1 h at 47 ℃. After digestion, the peptides were eluted with 40 µL of buffer A (5% aqueous acetonitrile with 0.1% formic acid), dried down, and resuspended in 20 µL buffer A. The reduced, alkylated and digested peptides were analysed by bottom-up proteomics analysis using an Ultimate 3000 RSLC nano ultra-high-performance liquid chromatography (nano UHPLC, Thermo Fisher Scientific, Waltham, USA) coupled to an Exploris 480 (Thermo Fisher Scientific, MA, USA) using nanoEase M/Z Peptide CSH C18 Column, 130 Å, 1.7 µm, 300 µm X 100 mm (Agilent Technologies, Santa Clara, USA).

### Data annotation of spatially resolved proteomics

Upon completion of the requisite cleansing procedures, trypsin is meticulously applied to the tissue surface, preceding the introduction of the matrix in the matrix-assisted laser desorption/ionisation MSI step. Subsequently, a peptide map is generated, facilitating the execution of direct MS^2^ fragmentation on the detected peptides in the bottom-up proteomics assay, thereby enabling the accurate identification of the associated proteins 18, 19. Quantile normalisation of the SWATH-MS data was performed before comparing groups. The approach employed in this study is based on the premise that peptide distributions within the specimens are comparable, with the mean of each quantile across the samples serving as a reference distribution for alignment purposes 20. Protein identification was carried out using the Homo sapiens taxonomic parameters in the Swiss-Prot database. Furthermore, high-confidence MS^2^ peptide fragments were utilised for MSI alignment within the SCILS LAB MVS (2022b) software (Bruker, Bremen, Germany). QVality q-value was used for nonparametric estimation of posterior error probabilities (PEP) 21 for peptide-protein annotation, and q-value < 0.01 was considered as high confidence. Detailed annotation is shown in **Table S3**.

### Bovine cartilage explant sample preparation

Fresh adult bovine knee joints (n = 3) were procured from the butcher for bovine explant research on the day of slaughter, as described previously 8. Full-thickness articular cartilage explants without the subchondral bone were extracted from the knee joints. Discs of cartilage were then dissected aseptically using a 4 mm dermal punch (Kai Medical, Gifu, Japan). The explants were grown in chondrogenic Eagle's minimal essential medium (DMEM), high (4.5 g/L) glucose (DMEM-HG, Invitrogen, Waltham, USA) supplemented with 1% inulin-transferrin-selenium (Sigma-Aldrich, MA, USA), 1.25 mg/mL bovine serum albumin (Sigma-Aldrich, St. Louis, USA), 0.1 μM dexamethasone (Sigma-Aldrich, St. Louis, USA), 0.1 mM ascorbic acid (Sigma-Aldrich, MA, USA), 1% penicillin-streptomycin, 10 mM hydroxyethyl piperazineethanesulfonic acid (HEPES) (Sigma-Aldrich, St. Louis, USA), 0.1 mM L-proline (Sigma-Aldrich, St. Louis, USA), 0.1 mM MEM nonessential amino acids (Thermo Fisher Scientific, Waltham, USA) and 10 ng/mL transforming growth factor-β1 (TGF-β1, Gibco, CA, USA)) supplemented with 10% fetal bovine serum (FBS) for 48 h at 37 ℃ and 5% carbon dioxide 22. The bovine cartilage explants were treated with or without Interleukin-1β (IL-1β) (10 ng/mL) for 7 days to mimic the OA phenotype 23.

### Immunohistochemistry

Immunohistochemistry was performed using previously described protocols 24. Briefly, samples were fixed overnight in 4% aqueous paraformaldehyde (PFA) and then decalcified using 10% aqueous ethylenediaminetetraacetic acid (EDTA) solution at 4 °C and exchanged every three to four days, resulting in a final pH of 7.2-7.4. Following decalcification, the samples were dehydrated, cleared, and impregnated with paraffin according to standard protocols using a Shandon Histocentre 3 Tissue Embedding Centre (Thermo Fisher Scientific, Waltham, USA). 10 μm sections were produced from paraffin blocks using a microtome, heated at 55 ℃, and deparaffinised using xylene and ethanol concentrations of 100%, 90%, and 70% 25. Antigen retrieval was conducted using proteinase K (Agilent Technologies, Santa Clara, USA), followed by the application of 3% aqueous hydrogen peroxide to the sections to suppress endogenous peroxidase activity. 2% bovine serum albumin was used to prevent nonspecific protein binding. The sections were treated progressively with primary and secondary EnVision Dual Connection-HRP antibodies and incubated in a 3,3'-Diaminobenzidine (DAB) solution (0.5 mL of 30% H_2_O_2_ in 1 mL DAB). The sections were then counterstained with Mayer's hematoxylin and dehydrated for 3 mins in 70%, 90%, 100%, and 100% ethanol and cleared with xylene, and then mounted with Entellan New mounting media before being scanned with a 3DHistech Scan II Brightfield slide scanner (3DHistech, Budapest, Hungary). Finally, the images were acquired by Slide Viewer 2.5 (3DHistech, Budapest, Hungary). The information on antibodies and dyes is included in **Table S2**.

### Data analysis

The statistical evaluation was performed using GraphPad Prism 8 software (GraphPad Software, San Diego, USA). To assess the normality of the data clusters, the Shapiro-Wilk test was implemented. All assessed data clusters successfully met the assumption of normality. Subsequently, a paired t-test was conducted to compare the variables of interest. Statistical significance was determined by p-values below 0.05 for the aforementioned processes. The findings of the study were presented as mean values along with standard deviations (SD).

## Results

### Biochemical and histopathological analysis

After harvesting femoral condyles subsequent to total knee replacement (**Figure 1A**), we conducted individual biochemical and histopathological assessments on 12 osteochondral units. The assessment focused on analysing proteoglycan (PG) loss through the utilisation of the Safranin-O/Fast green (saf-O) staining method. Notably, the saf-O staining exhibited lower PG loss in OA samples compared to non-OA samples, which were graded based on disease severity (**Figure 1B**). To classify the samples further, we employed the modified Mankin score using patient-matched sections from both medial and lateral femoral condyles based on saf-O staining (**Figure 1C**). Non-OA sections displayed a modified Mankin score of 2.50 ± 1.05, whereas OA sections exhibited a substantially higher score of 11.00 ± 4.55. The considerable difference in modified Mankin scores between OA and non-OA samples underscores the augmented severity of OA, as also evidenced by elevated PG loss in the OA group (*P* < 0.0001).

### Multiple elevated AA-containing phospholipids in the osteochondral unit during OA progression

**Figure 2:**
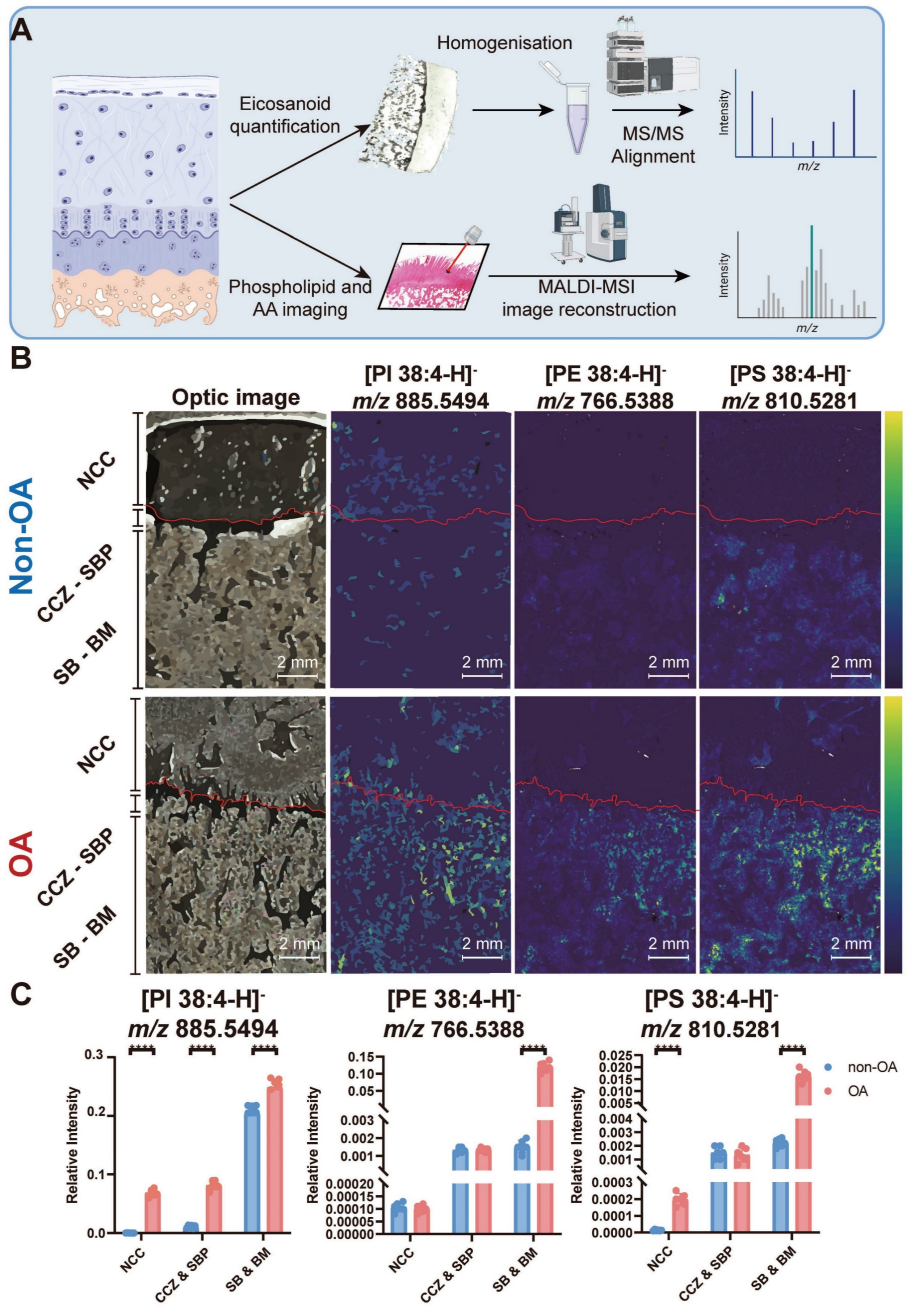
**Multiple AA-containing phospholipids change in the osteochondral unit during OA progression**. (**A**) Matrix-assisted laser desorption/ionisation mass spectrometry imaging (MALDI-MSI) workflow and MS^2^ alignment of AA-containing phospholipids in tissue sections. (**B**) MALDI-MSI analysis of AA-containing lipids in the human osteochondral unit. One non-OA and OA section from six different subjects were imaged, and one representative lesion of each type was shown. The leftmost images are representative of optical images of the tissues. (**C**) The quantification of arachidonic acid-containing phospholipids between non-OA and OA osteochondral units using MALDI-MSI divided according to stratigraphy. NCC: non-calcified cartilage; CCZ: calcified cartilage zone; SBP: subchondral bone plate; SB: subchondral bone; BM: bone marrow. Data are presented as means ± standard deviation (SD) for n = 6. Scale bar: 2 mm. *P* < 0.05 was considered significant. *P < 0.05, **P < 0.01, ***P < 0.001. The figure was created with BioRender.com.

The co-localisation of a substrate and product can serve as an indicator of specific enzyme activity. To test this, we used subjected sections of osteochondral tissue to MALDI-MSI to visualise changes in the relative abundance of multiple AA-containing phospholipids, including stearoyl-arachidonoyl-glycerophosphoinositol (PI 38:4), stearoyl-arachidonoyl-glycerophosphoethanolamine (PE 38:4) and stearoyl-arachidonoyl-glycerophosphoserine (PS 38:4) (**Figure 2A**). The identity of each lipid was confirmed by lipid extraction and nano-electrospray ionisation-tandem mass spectrometry (ESI-MS^2^, **Figure 2**, **Figure S1**). Integration of these lipid-specific signals from the MALDI-MSI measurements across different tissue zones reveals that in non-OA samples, PI 38:4 is more abundant in the area classified as subchondral bone (SB) and bone marrow (BM). Quantitively, the PI 38:4 relative intensity of non-calcified cartilage (NCC), calcified cartilage zone (CCZ) - subchondral bone plate (SBP), and subchondral bone (SB) - bone marrow (BM) were 2.0×10^-4^ ± 7×10^-5^, 1.1×10^-2^ ± 2×10^-3^ and 2.1×10^-1^ ± 0.6×10^-2^, respectively. In the non-OA sections, PI 38:4 localisation is more prevalent in OA BM, compared with CCZ -SBP and NCC. In the OA sections, the relative intensity of OA NCC, CCZ - SBP, and SB - BM were 6.9×10^-2^ ± 6×10^-3^, 8×10^-2^ ± 7×10^-3^, and 2.5×10^-1^ ± 0.7×10^-2^, respectively. OA sections have a significantly higher abundance compared with non-OA sections, especially in BM and NCC (P < 0.0001) (**Figure 2B-C**).

PE 38:4 is also more abundant in the SB - BM area in non-OA samples. Quantitively, the PE 38:4 relative intensity in NCC, CCZ - SBP, and SB - BM were 1.1×10^-4^ ± 2×10^-5^, 1.3×10^-3^ ± 2×10^-4^ and 1.5×10^-3^ ± 3×10^-4^, respectively. In the non-OA sections, PE 38:4 localisation is more prevalent in OA BM, compared with CCZ -SBP and NCC. In the OA sections, the relative intensity of OA NCC, CCZ - SBP, and SB - BM were 1.0×10^-4^± 1×10^-5^, 1.4×10^-3^ ± 1×10^-4^, and 1.2×10^-1^ ± 1×10^-2^, respectively. OA sections have a significantly higher abundance in OA sections compared with non-OA in BM and NCC (P < 0.0001) (**Figure 2B-C**).

In non-OA samples, PS 38:4 accumulates SB, SBP and CCZ regions. In terms of quantity, the relative PS 38:4 abundances of NCC, CCZ - SBP, and SB - BM were 1.3×10^-5^ ± 4×10^-6^, 1.5×10^-3^ ± 4×10^-4^ and 2.3×10^-3^ ± 3×10^-4^, respectively. In the non-OA sections, PS 38:4 localisation is more prevalent in OA BM than CCZ - SBP and NCC. In the OA sections, the relative intensity of PS 38:4 signals in OA NCC, CCZ - SBP, and SB - BM were 2.0×10^-4^ ± 4×10^-5^, 1.4×10^-3^ ± 5×10^-4^, and 1.7×10^-2^ ± 2×10^-3^, respectively. OA sections have a significantly higher abundance in OA sections compared with non-OA sections, especially in NCC (P < 0.0001), BM and SB (P < 0.0001) (**Figure 2B-C**). Overall, the PI 38:4, PE 38:4 and PS 38:4 share a similar distribution spatially across the osteochondral samples and are all elevated during OA progression.

### Elevated PLA_2_ activity in the human osteochondral unit during OA progression

**Figure 3:**
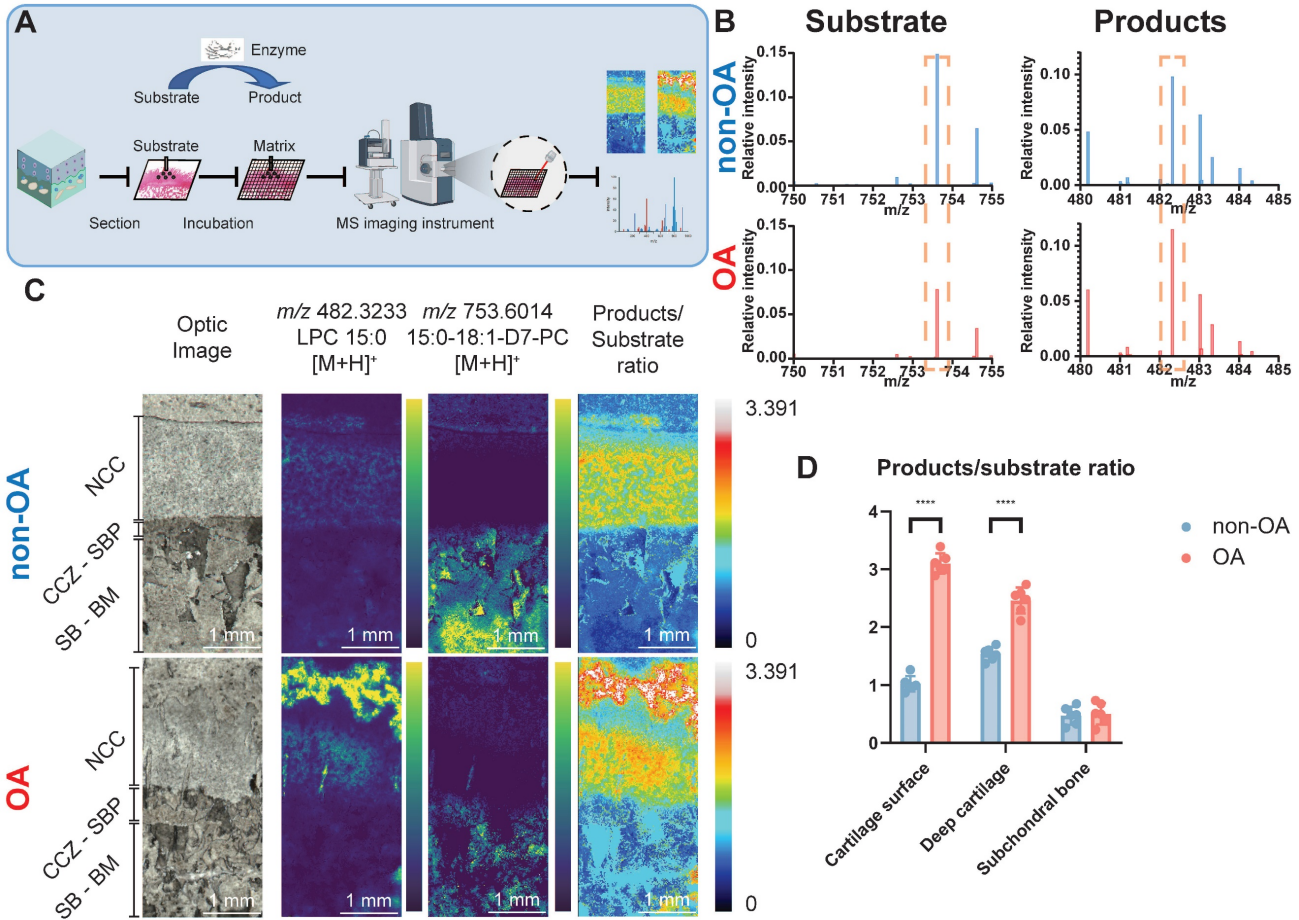
** Functional mass spectrometry (fMSI) of human non-OA and OA knee osteochondral unit**. (**A**) fMSI workflow. (**B**) Averaged MALDI mass spectrum of PC 15:0/18:1-D_7_ (enzyme-substrate) and LPC 15:0 (enzyme-products) in the absence of tissue. (**C**) MALDI-MSI ion abundance map of LPC 15:0, PC 15:0/18:1-D_7_ and LPC 15:0-to-PC 15:0/18:1-D_7_ ratio from human osteochondral tissue. One non-OA and OA section from six different subjects were imaged, and one representative lesion of each type was shown. (**D**) The relative intensity ratio of LPC 15:0-to-PC 15:0/18:1-D_7_ between non-OA and OA osteochondral units using MALDI-MSI divided according to stratigraphy. NCC: non-calcified cartilage; CCZ: calcified cartilage zone; SBP: subchondral bone plate; SB: subchondral bone; BM: bone marrow. Data are presented as means ± standard deviation (SD) for n = 6. Scale bar: 1 mm.* P* < 0.05 was considered significant. *P < 0.05, **P < 0.01, ***P < 0.001. The figure was created with BioRender.com.

fMSI experiments were conducted to establish whether the higher abundance of the three AA-containing phospholipids is correlated with each other and to determine the biological enzyme activity that occurred during OA progression. To achieve fMSI analysis, we applied glycerophospholipid substrates to tissue sections to map PLA_2_ activity directly off the human osteochondral interface that had been graded according to disease severity (**Figure 3A**). The results (**Figure 3B-D**) revealed that product ions resulting from phospholipase activity ([LPC 15:0+H]^+^, *m/z* 482.3233) were present in the OA cartilage surface and deep cartilage layer but with notable regional variation, such as lower abundance and even small patches devoid of PLA_2_ activity in SBP, SB, and BM. In contrast, the distribution of intact substrate ([PC 15:0/18:1-D7+H]^+^, *m/z* 730.6014) was found to be primarily restricted to locations such as BM, where we found only an extremely low LPC signal, corresponding to a small amount of MALDI-induced LPC production. The results reflect the elevated proinflammatory AA-containing phospholipids distribution with the distribution of PLA_2_ activity in OA samples.

### Elevated PLA_2_ activity in bovine cartilage explant OA mimic sample

**Figure 4:**
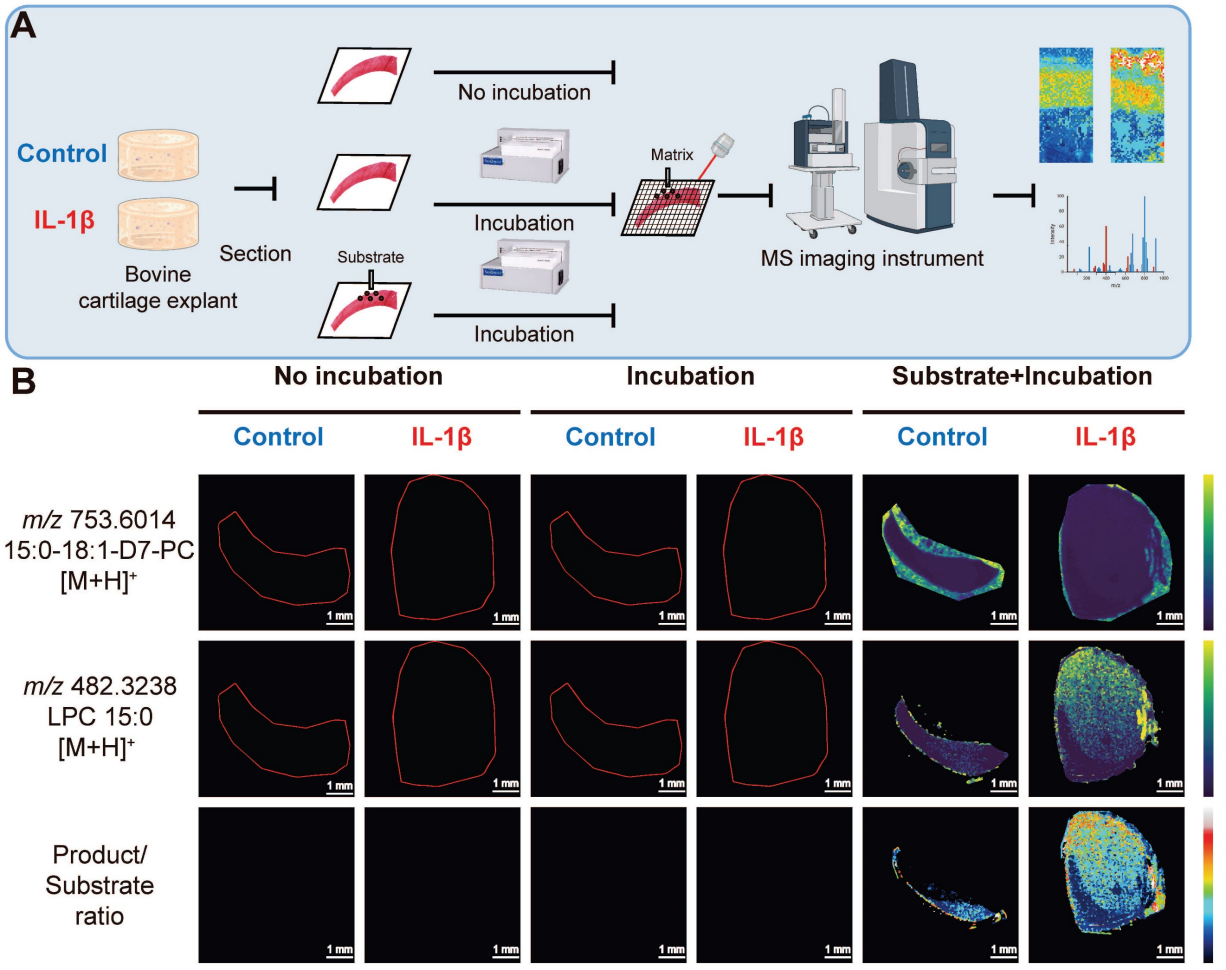
** Functional mass spectrometry (fMSI) of non-OA and OA bovine cartilage explant**. (**A**) Bovine explant fMSI workflow. (**B**) MALDI-MSI ion abundance maps of PC 15:0/18:1-D_7_ (top), LPC 15:0 (mid), and the ratio between LPC 15:0 and PC 15:0/18:1-D_7_ (bottom) for the negative control and interleukin-1 beta (IL-1β) OA mimic bovine explants. Three non-OA and OA sections from two groups in three experimental settings were imaged, and one representative lesion of each type was shown. Data are presented as sample mean ± standard deviation (SD) for n = 3. Scale bar: 1 mm. The figure was created with BioRender.com.

To determine whether OA increased PLA_2_ activity, we employed fMSI on a bovine cartilage explant-OA mimic sample (**Figure 4A**). We observed (**Figure 4B**) that product ions ([LPC 15:0+H]^+^, *m/z* 482.3233) resulting from phospholipase activity on the deposited PC 15:0/18:1-D_7_ were present in the subtrate+incubation+IL-1β treated group but were absent in the no-incubation group and the incubation-only group, thus revealing a lack of PLA_2_ activity. In contrast, the distribution of intact substrate ([PC 15:0/18:1-D_7_+H]^+^, *m/z* 730.6014) is predominantly restricted to the incubation and the control group, where we detected only a negligible LPC signal likely stemming from source fragmentation of the diacyl PC standard. Regardless of whether they were incubated or not, the tissues not sprayed with the deuterated PC standard exhibited no signal intensity that covered the spectrum of PC 15:0/18:1-D_7_ and LPC 15:0.

### Phospholipase A2 group IIA (PLA_2_G_2_A) is responsible for PLA_2_ enzyme activity change at the tissue level during OA progression

**Figure 5:**
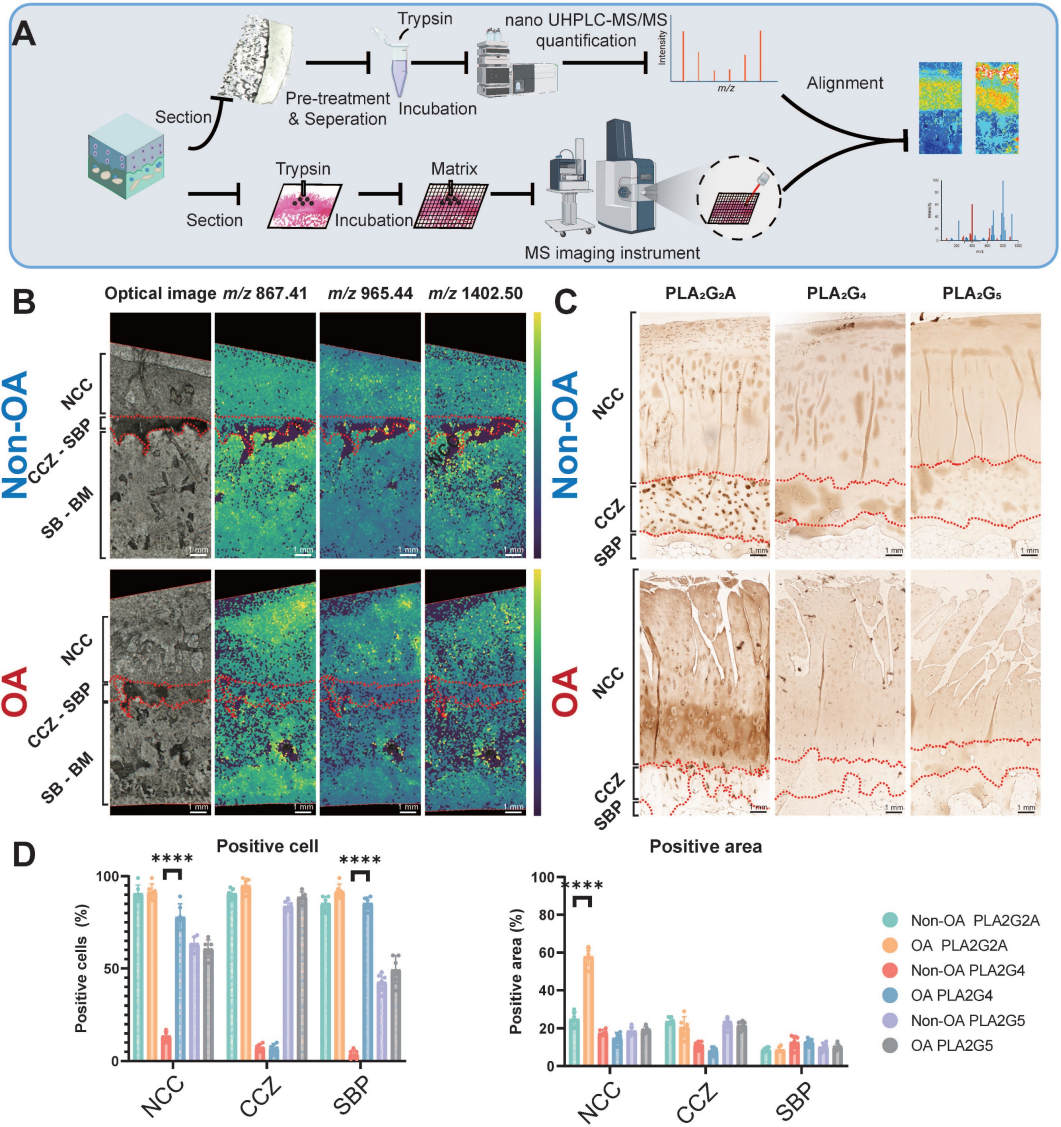
** Validation of functional mass spectrometry imaging (fMSI) by spatially resolved proteomics and immunohistochemistry (IHC)**. (**A**) Peptide mass fingerprinting and immunohistochemistry workflow. (**B**) MALDI-MSI abundance maps displaying nanoscale high-performance liquid chromatography coupled to mass spectrometry (nano UHPLC-MS) ion channels aligned to the PLA_2_G_2_A MSI in a comparison between non-OA and OA osteochondral units. (**C**) Representative IHC analysis for non-OA and OA osteochondral units using human knee samples. One non-OA and OA section from six subjects were imaged, and one representative lesion of each type was shown. (**D**) NCC: non-calcified cartilage; CCZ: calcified cartilage zone; SBP: subchondral bone plate; SB: subchondral bone; BM: bone marrow. Data are presented as means ± standard deviation (SD) for n = 6. Scale bar: 1 mm.* P* < 0.05 was considered significant. *P < 0.05, **P < 0.01, ***P < 0.001. The figure was created with BioRender.com.

Since various PLA_2_ enzyme subtypes exert the same enzyme activity within the tissue but have distinct distributions, it is crucial to determine the specific enzyme subtype that changes the enzyme activity at the tissue level. We employed peptide mass fingerprinting, aligning tandem MS with trypsin-digested MSI, for untargeted protein identification (**Figure 5A-B**). We also employed IHC to three subtypes of PLA_2_, previously reported to change during OA progression, to support our findings 26. Our spatially-resolved proteomics results showed that PLA_2_G_2_A increases in the superficial and deep cartilage layers. Our immunohistochemistry analyses further displayed that PLA_2_G_2_A was less abundant in non-OA NCC (**Figure 5C-D**) but more abundant in the OA cartilage surface and deep cartilage zone. Concurrently, PLA_2_G_4_ displayed a more positive cartilage cell rate in the OA tissue, whereas we found no difference in PLA_2_G_5_ when comparing non-OA and OA sections. All the above findings indicate that the PLA_2_G_2_A is responsible for PLA_2_ enzyme activity change at the tissue level during OA progression.

## Discussion

Previous studies have focused on analysing the bulk proteomic, metabolomic, and lipidomic differences between healthy and OA cartilage, synovium and synovial fluid samples 23, 27-30. However, these traditional approaches primarily assess protein abundance or gene expression and may not fully capture the functional state of specific biomolecules, such as enzymes, within the tissue. Here, we developed fMSI to directly investigate the functional aspects of enzymes in the context of OA. This innovative approach provides an additional layer of information, enabling us to gain insights into the molecular alterations and functional changes associated with OA. To the best of our knowledge, this is the first study to apply fMSI to osteoarthritic tissues, aiming to elucidate the links between the lipidome and proteome and visualise spatial inflammation mediators during the progression of OA.

**Figure 6:**
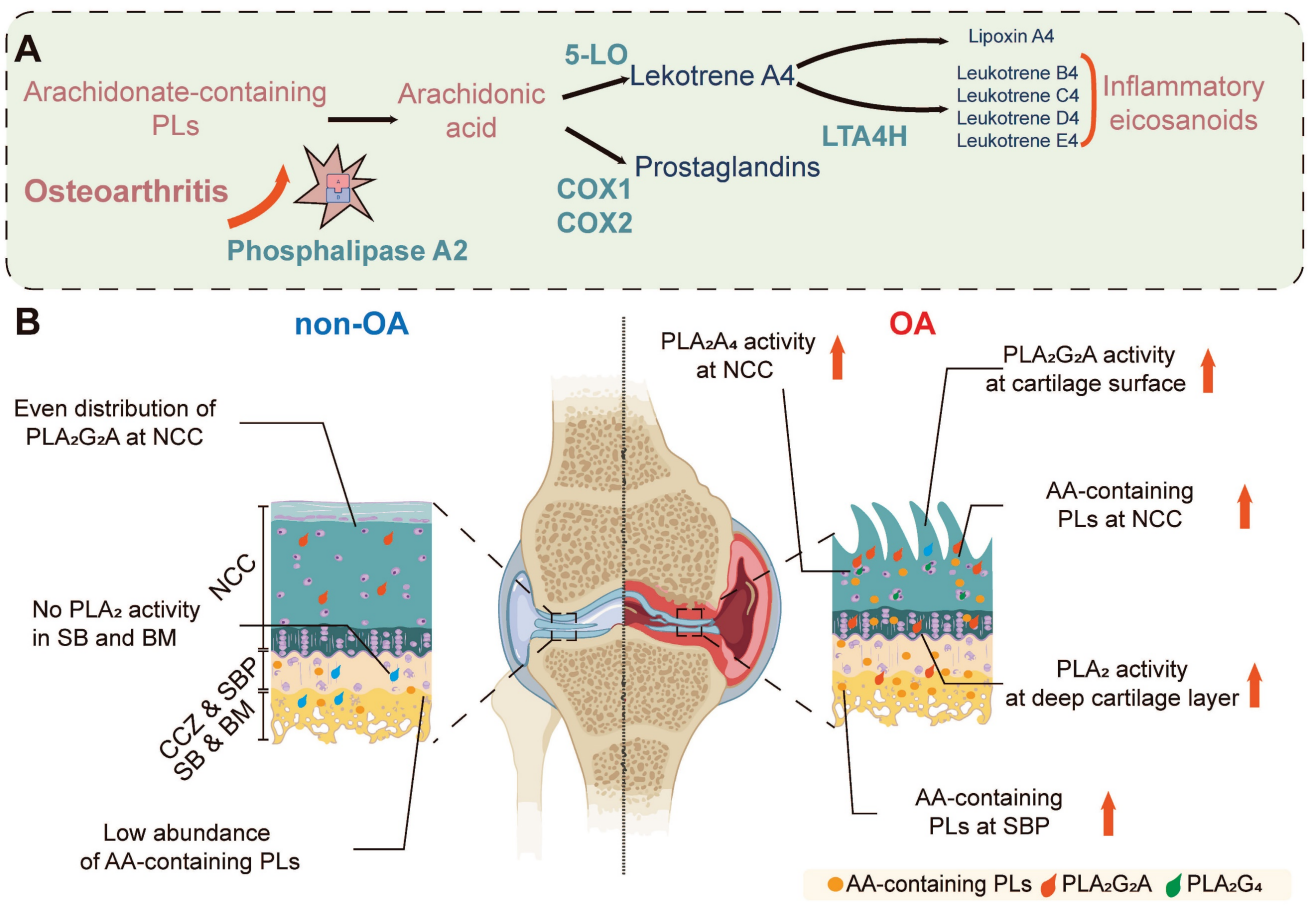
** Spatial PLA_2_ activity change and spatial AA metabolism change during OA progression**. (**A**) OA leads to increased PLA_2_G_2_A in the osteochondral unit, which goes on to release stored AA from phospholipids which would subsequently cause the increased excitation of downstream proinflammatory signalling pathways. (**B**) In the normal osteochondral unit, minimal PLA_2_ activity is observed in the cartilage layer, and a relatively low abundance of AA-containing phospholipids locate in the BM. However, during OA progression, PLA_2_G_2_A increases in the superficial and deep layer cartilage, and PLA2G4 increases in the chondrocytes. The AA-containing phospholipids increase in the BM and cartilage. AA: arachidonic acid; NCC: non-calcified cartilage; CCZ: calcified cartilage zone; SBP: subchondral bone plate; BM: bone marrow; PLs: Phospholipids; 5-LO: 5-Lipoxygenase; COX-1: cyclooxygenase-1; COX-2: cyclooxygenase-2; LTA4H: leukotriene A4 hydrolase. The figure was created with BioRender.com.

In this study, several enzymatic biochemical changes have been identified in OA disease progression, including an increase in multiple AA-containing phospholipids (*P* < 0.001) and the increase is observed in both the surface and deep layers of OA cartilage, which is associated with elevated PLA_2_ activity (*P* < 0.001). Bovine explant tissues treated with IL-1β were used to validate these findings to simulate OA pathophysiology. These samples displayed elevated levels of PLA_2_ compared to controls (*P* < 0.001), confirming the role of PLA_2_ in OA pathology and validating our technique and the importance of this enzyme. Further investigation revealed that the PLA_2_G_2_A isoform is specifically responsible for the observed changes in PLA_2_ enzyme activity in OA cartilage tissues. These findings highlight the distinct distribution of PLA_2_ enzyme activity throughout the progression of OA. We also observed an elevation of proinflammatory substrates in the OA NCC and SB, as indicated by multiple AA-containing phospholipids in these regions and changes in their relative abundance during OA progression. A summary of the observed differences is illustrated in **Figure 6**.

In line with our studies, Ciellero-pastor 31 utilised MALDI-MSI to identify protein changes in PLA_2_ within diabetic OA samples. However, our study goes beyond protein expression and focuses on demonstrating the functional enzyme activity state, which is often considered more important than protein expression because it directly reflects the functional state within the tissue. Specifically, we conducted downstream validation using OA samples graded according to the disease severity, providing valuable insights into the enzymatic alterations occurring in patients and also can mimic this in vitro in explant cultures under inflammatory conditions. At the spatial level, we found that the enzymatic activity of PLA_2_G_2_A is upregulated not only in the deep layer of the cartilage but also damaged cartilage surface. In our study, we used a new tissue preparation approach that effectively preserved both the morphological integrity of the tissue and the spatial distribution of enzymatic activity. In contrast to conventional methodologies involving the separation of cartilage layers from the bone, which may compromise tissue integrity and spatial information, our method enabled the retention of the spatial context of mineralised tissue while facilitating in situ detection of enzymatic activity.

Lipid-related inflammation pathways are deeply involved in OA progression. Previous studies have shown that increased COX1, COX2 expression and elevated PGE2 levels in the cartilage are linked to the pathological changes observed in OA 5. In this study, we observed variable changes in levels and spatial distributions of a variety of lipid proinflammatory mediators related to lipids homeostasis, including multiple AA-containing phospholipids like PI 38:4, PE 38:4 and PS 38:4. PI 38:4 is the most abundant PI in normal tissues 32. AA-containing phospholipids are also involved in various inflammatory diseases, including tuberculosis 26 and colorectal cancer 33. PI 38:4 is subjected to the Lands cycle, whereby PI 38:4 is converted into AA and LPI by PLA_2_. Diacyl PI can then be regenerated by incorporating the fatty acid, including AA, into the *sn-*2 position of LPI by lysophosphatidylinositol acyltransferase 1 (LPIAT1, also known as MBOAT7). Importantly, PI 38:4 also serves as a substrate for phosphatidylinositol polyphosphates (PIPs), which play vital roles in a wide range of cellular processes, including cell migration, invasion, and proliferation 34. Given the observed variations in PI, AA, and PLA_2_ distribution throughout osteoarthritic tissues, it is reasonable to suggest that the inflammatory effects of PIPs may contribute to OA development.

Our study also demonstrated the robustness, versatility, and sensitivity of fMSI among different species. fMSI was first reported as a methodology for PLA_2_ enzyme activity distribution using the brown forest cobra (*Naja. subfulva*) as the animal model 1 since the venom gland contains abundant PLA_2_ enzyme that is even active after fixing for formalin-fixed paraffin-embedded (FFPE) sections. However, whether fMSI works for tissues such as bone and cartailge with a lower abundance of active enzymes remained uncertain. This work has successfully validated the fMSI approach using two different non-PLA_2_-rich tissue that involves PLA_2_ as the target enzyme system, indicating that this technique is a robust protocol that can be implemented across various species (human and bovine), provided specific enzyme/substrate combinations are identified.

Regarding the substrate-enzyme-products relationship, a comparison of the distribution of LPC 15:0 in the OA tissue of **Figure 3C** and the darker regions in the OA tissue IHC of PLA_2_G_2_A in **Figure 5C** confirms that PLA_2_ is indeed present and active in the specific regions of OA NCC. Additionally, **Figure 2B** shows a moderate increase of multiple AA-containing phospholipids in OA NCC. Thus, the results confirm that substrates and downstream metabolites reflect enzyme activity and tissue dispersion. The result where AA-containing phospholipids appear absent from the superficial layer is exceptional as it suggests the involvement of biochemical pathways that were not monitored in this study. Various explanations could be responsible for this result, including AA being a substrate for numerous molecules of the cyclooxygenase (COX) and lipoxygenase (LOX) eicosanoid pathways, lyso-lipid reformation into non-AA containing phospholipids, such as PC 36:1, which is present in the superficial layer, or involvement of lyso-lipid species in other signalling cascades. These findings align with the enzymatic observations made in our study.

The fMSI method developed in this study holds promise for clinical applications, particularly in the context of OA and other joint diseases such as Rheumatoid Arthritis. Its unique ability to visualise and map enzymatic activity within tissues provides valuable insights into disease-associated enzymes' spatial distribution and functional characteristics 1, 3. This precise mapping of enzymatic activity enhances our understanding of disease mechanisms and aids drug development, biomarker discovery, and identification of potential therapeutic targets. Additionally, fMSI's capability to monitor enzymatic changes over time allows for evaluating treatment efficacy and enables personalised precision medicine approaches based on patients' unique enzymatic profiles, optimising treatment outcomes and reducing adverse effects.

However, some limitations need to be addressed to optimise the potential of fMSI for future clinical applications. Firstly, the relatively lower spatial resolution of fMSI compared to alternative imaging approaches like IHC can hinder data resolution. Fine-tuning the imaging parameters, such as timeframe and temperature, is essential to enhance the noise-signal ratio and improve data quality. Another limitation lies in the potential occurrence of ion suppression effects during fMSI, compromising confidence in molecular identification. Interference from endogenous molecules and matrix components in complex tissue samples can reduce target analytes' signal intensities, affecting molecular identification and data interpretation. Furthermore, the technical expertise, associated prolonged imaging times, data analysis, and high costs required for fMSI may limit its accessibility in specific settings. By optimising protocols, standardising procedures, and tackling practical challenges, fMSI can become a valuable tool for advancing our understanding of biological processes and disease mechanisms in clinical settings.

## Conclusion

To conclude, we demonstrate the application of the fMSI technique to disease-state models to gain a greater understanding of the spatial aspects of OA pathogenesis. The current research also indicates that endogenous substrates and related products do not reflect the abundance of enzyme activity, thus indicating the necessity of a non-endogenous substrate to map enzyme activity using fMSI confidently. Although our results require confirmation in larger sample sets, the data presented suggest that the local changes in metabolites and enzyme activity are equally crucial in OA. It also establishes the foundation for future research on the spatial distribution of OA to ascertain its relationship to disease progression and its potential contribution to treatments that may slow or even reverse the progression of the disease.

## Supplementary Material

Supplementary figure and tables.

## Abbreviations

OA: osteoarthritis
MSI: mass spectrometry imaging
fMSI: functional mass spectrometry imaging
IHC: immunohistochemistry
MALDI-MSI: matrix-assisted laser desorption/ionisation mass spectrometry imaging
PLA_2_: phospholipase A2
AA: arachidonic acid
TOF: time-of-flight
BMI: body mass index
SCEM: super-cryo embedding medium
TIC: total ion current
ROI: regions of interest
MS^1^: mass spectrometry
PEP: posterior error probabilities
MS^2^: tandem mass spectrometry
PC: phosphatidylcholine
PC O-: ether-linked phosphatidylcholine
PE: phosphatidylethanolamine
PE O-: ether-linked phosphatidylethanolamine
PS: phosphatidylserine
PG: phosphatidylglycerol
PI: phosphatidylinositol
SM: sphingomyelin
LPC: lysophosphatidylcholine
LPE: lysophosphatidylethanolamine
LPS: lysophosphatidylserine
LPG: lysophosphatidylglycerol
LPI: lysophosphatidylinositol
MTBE: methyl-tert-butyl ether extraction
SDS: sodium dodecyl sulphate
DTT: dithiothreitol
IAA: iodoacetamide
ABC: Ammonium bicarbonate
nano UHPLC: nano ultra-high-performance liquid chromatography
DMEM: Eagle's minimal essential medium
HEPES: hydroxyethyl piperazineethanesulfonic acid
TGF-β1: transforming growth factor-β1
FBS: fetal bovine serum
EDTA: ethylenediaminetetraacetic acid
DAB: 3,3′-Diaminobenzidine
SD: standard deviations
ESI-MS^2^: lipid extraction and nano-electrospray ionisation-tandem mass spectrometry
NCC: non-calcified cartilage
CCZ: calcified cartilage zone
SBP: subchondral bone plate
SB: subchondral bone
BM: bone marrow
IL-1β: Interleukin-1β
PLA_2_G_2_A: phospholipase A2 group IIA
LPIAT1: lysophosphatidylinositol acyltransferase 1
PIPs: phosphatidylinositol polyphosphates
FFPE: formalin-fixed paraffin-embedded
COX: cyclooxygenase
LOX: lipoxygenase
