# Supplementary material for: Functional mass spectrometry imaging maps phospholipase-A2 enzyme activity during osteoarthritis progression
Source: Theranostics. 2023 Aug 21;13(13):4636–49. doi: 10.7150/thno.86623 (PMC10465221; doi:10.7150/thno.86623)
Supplement: Supplementary file 1 — Supplementary figure and tables. [file thnov13p4636s1.pdf]

Supplementary figure

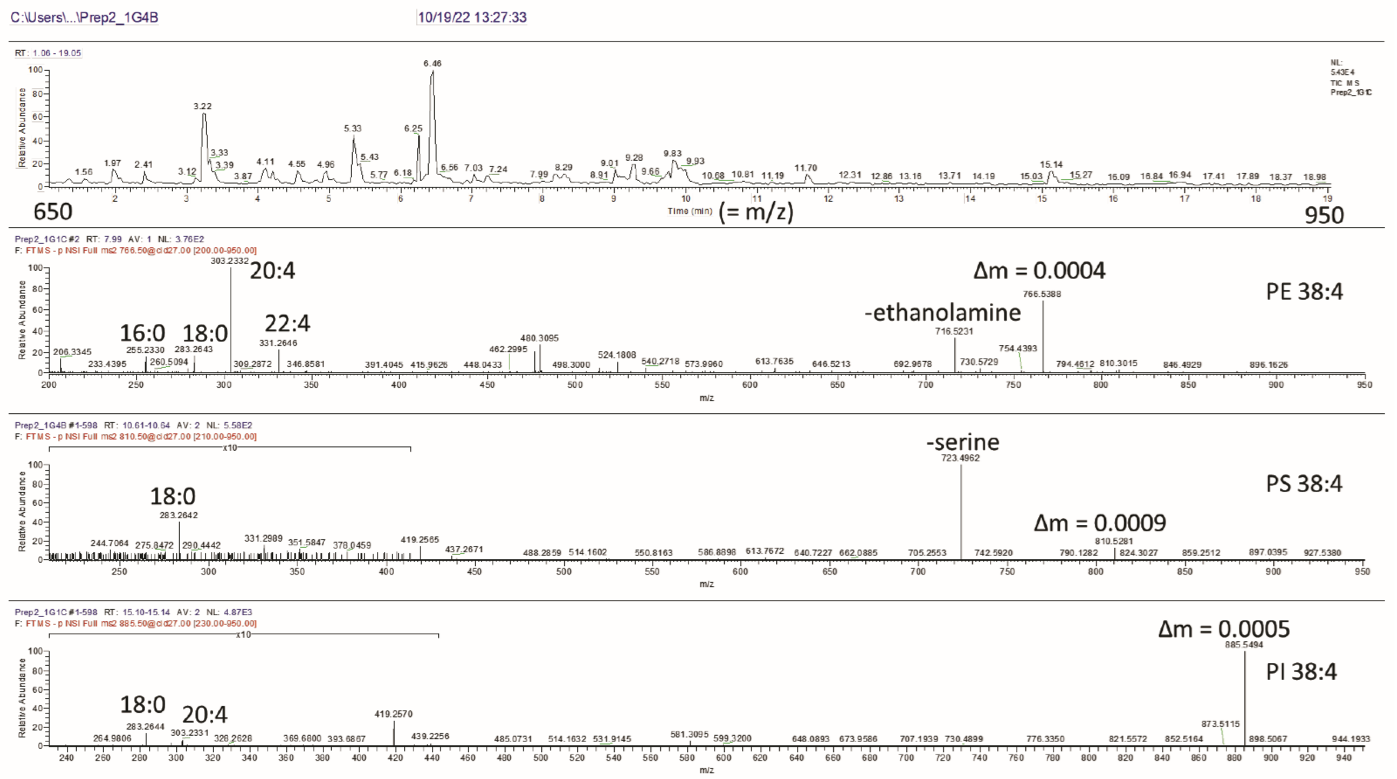

Figure S1. **Annotation of stearoyl-arachidonoyl-glycerophosphoinositol (PI 38:4), stearoyl-arachidonoyl-glycerophosphoethanolamine (PE 38:4) and stearoyl-arachidonoyl-glycerophosphoserine (PS 38:4) based on electrospray ionisation tandem mass spectrometry (ESI-MS/MS)**. Three arachidonic acid-containing phospholipids, including PI 38:4, PE 38:4 and PG 38:4, were annotated from the human osteochondral unit.

Supplementary tables

Table S1. Demographic information of patients enrolled in the study

No	Age	Gender	Weight (kg)	Height (cm)	BMI ($kg \cdot m^{-2}$)
1	70	Female	73	158	29.2
2	73	Female	74	164	27.5
3	84	Male	113	178	35.7
4	66	Female	88	163	33.1
5	77	Male	90	169	31.5
6	75	Male	87	178	27.5

Table S2. Antibodies and dyes

Primary antibodies	Species	Source (Catalog and clone number)	Research Dilution
PLA_2G_2A	Rabbit	Thermo Fisher Scientific (PA5-102403)	1:200
PLA_2G_4A	Rabbit	Thermo Fisher Scientific (PA5-75182)	1:200
PLA_2G_5	Rabbit	Thermo Fisher Scientific (PA5-84150)	1:200

Highly cross-absorbed secondary antibodies for IHC	Species	Source (Catalog and clone number)	Research Dilution
EnVision Dual Link System - HRP	Goat	Agilent Technologies (K406189-2)	Ready-to-use

Other reagents for IHC	Source (Catalog and clone number)	Research Dilution
Proteinase K	Agilent Technologies (S302030-2)	Ready-to-use

Table S3. Proteins identification from the non-OA or in OA cartilage

**Cartilage Swiss-Prot accession number**	**Protein name**	**Observed mass/charge ratio**	**Peptide sequence**	**Modifications**	**Qvality q-value**
A0A3B3IRX2	Phospholipase A2 Group IIA	867.4142	AAATCFAR	1xCarbamidomethyl [C5]	0.002116
		965.4363	YQYYSNK	2xCarbamidomethyl [C4; C6]	0.002116
		1402.501	CCVTHDCCYK	4xCarbamidomethyl [C1; C2; C7; C8]	0.000385
